# Angiogenesis Inhibitors as Anti-Cancer Therapy Following Renal Transplantation: A Case Report and Review of the Literature

**DOI:** 10.3390/curroncol28010064

**Published:** 2021-01-22

**Authors:** Lawrence Kasherman, Jeffrey Doi, Katherine Karakasis, Jeffrey Schiff, Abhijat Kitchlu, Stephanie Lheureux, Amit M. Oza

**Affiliations:** 1 Division of Medical Oncology & Hematology, Bras Family Drug Development Program, Princess Margaret Cancer Centre, University Health Network, University of Toronto, Toronto, ON M5G2M9, Canada; Lawrence.Kasherman@health.nsw.gov.au (L.K.); Katherine.Karakasis@Uhn.ca (K.K.); Stephanie.lheureux@uhn.ca (S.L.); 2 Department of Pharmacy, Princess Margaret Cancer Centre, University Health Network, University of Toronto, Toronto, ON M5G2M9, Canada; jeffrey.doi@uhn.ca; 3 Toronto Transplant Institute, University Health Network, University of Toronto, Toronto, ON M5G2C4, Canada; jeffrey.schiff@uhn.ca; 4 Division of Nephrology, University Health Network, University of Toronto, Toronto, ON M5G2C4, Canada; Abhijat.Kitchlu@uhn.ca

**Keywords:** bevacizumab, vascular endothelial growth factor, angiogenesis inhibitor, transplant, proteinuria, nephrotoxicity, case report

## Abstract

Solid organ transplant recipients on long-term immunosuppressive medication are at increased risk of developing malignancy, and treatment of advanced cancers with angiogenesis inhibitors in this context has not been widely studied. We present a case of recurrent high-grade serous ovarian carcinoma treated with paclitaxel and bevacizumab in the context of prior renal transplantation where the patient responded well to treatment with controlled toxicities, discussing the potential for increased rates of adverse events and drug interactions in this select population.

## 1. Introduction

**Figure 1:**
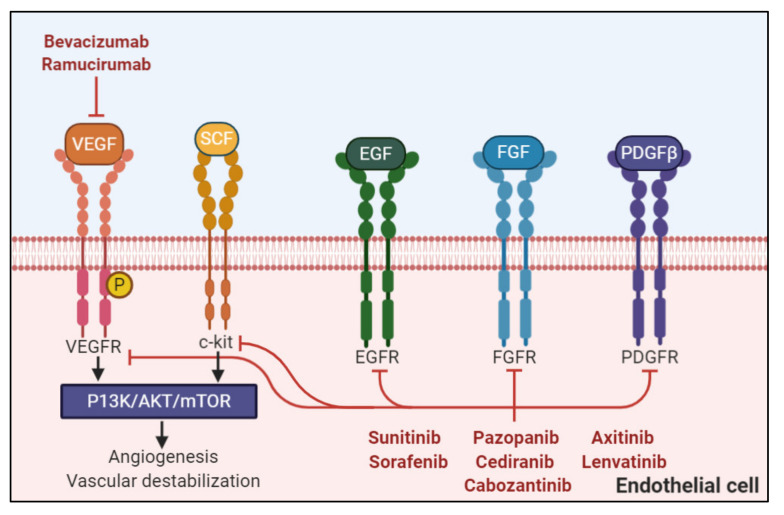
Angiogenesis pathways in malignancy and targeted therapies commonly used. Monoclonal antibodies including bevacizumab and ramucirumab and tyrosine kinase inhibitors including sunitinib, sorafenib, lenvatinib, pazopanib, axitinib, cediranib, and cabozantinib have been included (this list is not exhaustive). Receptors including VEGFR, c-kit, epidermal growth factor receptor (EGFR), fibroblast growth factor receptor (FGFR), and PDGFR are displayed here, but certain medications also target other pathways not displayed here such as AXL and RET. The VEGFR pathway intersects with multiple cell signaling pathways, including the PI3K/AKT/mTOR pathway. Created with Biorender.com.

Angiogenesis inhibitors such as vascular endothelial growth factor (VEGF) monoclonal antibodies and tyrosine kinase inhibitors (Figure 1) are standard treatments across various cancer subtypes. In advanced high-grade serous ovarian cancer (HGSOC), bevacizumab is part of the standard of care as maintenance therapy in front-line and recurrent disease [1]. Although the toxicity profile is generally well-managed, nephrotoxicity manifesting as proteinuria remains an important adverse event that requires close monitoring [2]. There is limited literature surrounding angiogenesis inhibitors as anti-cancer treatment in patients who have received solid organ transplants, and thus this report presents a case demonstrating safety of angiogenesis inhibition as anti-cancer therapy in a patient with a stable renal transplant.

## 2. Case Description

Consent: Fully informed, voluntary, written consent has been obtained to include patient information and publish this report.

Ethics: As this is a case report with fewer than three patients, institutional approval was not sought as per the University Health Network Research Ethics Board guidance document on case reports.

**Figure 2:**
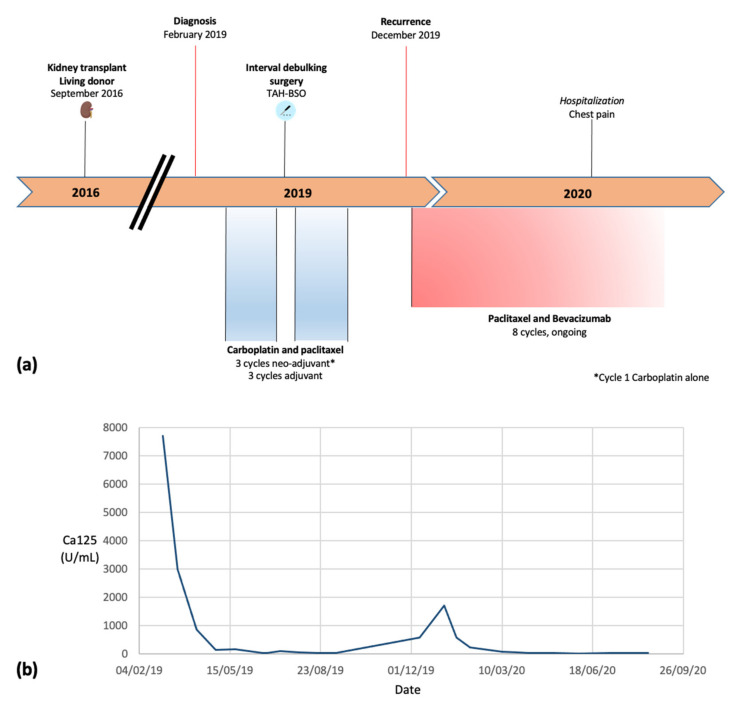
(**a**) Summary oncology treatment timeline. (**b**) Ca125 trend from diagnosis to current.

A 47-year-old woman presented with several months of dyspnea and abdominal distension to Princess Margaret Cancer Centre in February 2019 (Figure 2). CT revealed a 13.9 cm pelvic mass with peritoneal carcinomatosis and ascites. Omental biopsy confirmed HGSOC, and she received neoadjuvant platinum-based chemotherapy with excellent tolerance and no renal complications before proceeding to interval debulking in June 2019. There was no visible residual disease, and diagnosis of HGSOC was confirmed, germline and somatic *BRCA* wild type. She completed three cycles of adjuvant chemotherapy.

Her background was significant for IgA nephropathy, which resulted in progressive chronic kidney disease for 18 years prior to living donor kidney transplant in 2016. Both she and her donor were cytomegalovirus-positive, and she developed cytomegalovirus-associated colitis shortly post-transplantation. Her initial immunosuppression consisted of basiliximab induction followed by tacrolimus, mycophenolic acid, and steroids. She developed antibody-mediated rejection one week post-transplant, which was treated with plasmapheresis, immunoglobulin, and an increase in steroid dose. This was repeated three months later due to biopsy confirming ongoing antibody-mediated rejection. Mycophenolic acid was stopped upon HGSOC diagnosis. She has remained medication-adherent, with regular serum tacrolimus levels within the target range (most recently, 5.3 micrograms/L) and no signs of chronic graft rejection.

Her other comorbidities include diet-controlled, steroid-induced diabetes mellitus; ductal breast carcinoma in situ requiring wide local excision in 2011; asthma; reflux disease; hypertension; and hyperlipidemia. Other medications include prednisone 5 mg daily, acetylsalicylic acid, bisoprolol, trimethoprim–sulfamethoxazole, vitamin D, and inhaled salbutamol as needed.

In December 2019, she developed recurrence in the peritoneum and retroperitoneal lymph nodes, signifying platinum resistance. In January 2020, she commenced weekly paclitaxel 80 mg/m^2^ with bevacizumab 10 mg/kg every two weeks, and at that time, tacrolimus dose was reduced to 2 mg daily to aim for a serum level of 5 micrograms/L. She continued this therapy for over 6 months, and continued on the same dose of immunosuppression throughout with tacrolimus levels ranging between 3 and 7.6 micrograms/L. Serial imaging and Ca125 confirmed good response to treatment with reduction in size of tumor deposits. Her albumin/creatinine ratio was normal at 0.9 prior to the diagnosis of ovarian cancer, and most recently has been 15.6, signifying microalbuminuria; this has been monitored via urinalysis, which has consistently reported protein as negative or trace. Her most recent estimated glomerular filtration rate was 55 mL/min, similar to pre-diagnosis, and creatinine levels have mostly fluctuated between 95 and 120 umol/L.

Her course has been complicated by grade 2 hypertension (up to 145/95 mmHg) and non-cardiac chest pain, for which amlodipine was switched to ramipril 10 mg daily, and bevacizumab was withheld on two different occasions. Furthermore, she developed a brief period of Kidney Disease: Improving Global Outcomes (KDIGO) stage 1 acute kidney injury (AKI) of pre-renal etiology in September 2020 with creatinine of 160 umol/L, which self-resolved following withdrawal of bevacizumab, and was resumed after a 1-month-long break with no further episodes of kidney injury.

## 3. Discussion

Nephrotoxicity with angiogenesis inhibitors is relatively common, with proteinuria occurring in over 60% of patients [2]. Most cases are low-grade, transient, and do not require interventions or dose delays; however, more persistent, severe cases presenting as AKI and nephrotic syndrome can occur [2,3]. Risk factors associated with high-grade proteinuria include increased dose, prolonged administration, pre-existing renal disease, and administration of concurrent chemotherapy [4,5].

The pathophysiology of VEGF inhibitor-induced proteinuria remains unclear. Within a normal kidney, VEGF is produced by podocytes, and VEGF receptors are typically present on the glomerular and peritubular endothelium in addition to mesangial cells [6]. Inhibition of VEGF is thought to cause loss of endothelial fenestrations, podocyte injury and reduce endothelial proliferation, ultimately causing disruption of glomerular membranes [6]. Some cases have also demonstrated subacute thrombotic microangiopathy with endotheliosis and membranoproliferative changes [7]. Another manifestation of nephrotoxicity that is commonly seen is hypertension, occurring in more than a third of patients, which arises due to various mechanisms of renal vascular injury including inhibition of nitric oxide, rarefaction of microvasculature, and neuroendocrine dysregulation [8,9]. It is also hypothesized to increase intraglomerular pressure and ultrafiltration, leading to proteinuria [10].

The lack of clarity surrounding pathophysiology of VEGF inhibitor-induced nephrotoxicity [11] is demonstrated by the heterogeneity of published reports on glomerulopathy and other manifestations, including minimal change disease, collapsing glomerulopathy, membranoproliferative glomerulonephritis, focal segmental glomerulosclerosis, cryoglobulinemic glomerulonephritis, acute tubular necrosis, and interstitial nephritis [7]. Furthermore, worsening kidney disease can further exacerbate hypertension, which may perpetuate AKI [10].

Treatment for low-grade proteinuria usually includes an angiotensin-converting enzyme (ACE) inhibitor or an angiotensin receptor blocker to reduce glomerular filtration pressure, and anti-VEGF treatment can be continued providing stable proteinuria. However, proteinuria may worsen to the nephrotic range (>3 g in 24 h) with nephrotic syndrome. Even after cessation of VEGF inhibitor therapy, there are documented cases of persistent proteinuria [12].

### 3.1. Angiogenesis Inhibitors Post-Transplant

The role of the VEGF pathway in the pathogenesis of post-transplant complications is poorly understood, with preliminary reports across various organ transplants showing hypothesis-generating results [13,14,15,16]. Upregulation of angiogenesis factors was associated with increased allograft vasculopathy, bronchiolitis obliterans, and recurrence of hepatocellular carcinoma in cardiac, pulmonary, and liver transplants, respectively [13,15,16,17]. In renal transplants, VEGF is thought to be upregulated in acute and chronic rejection, particularly associated with cyclosporine [14].

As the first VEGF inhibitor used for anti-cancer treatment, bevacizumab was approved by the United States Food and Drug Administration in 2004, and is now licensed for use in many cancers [18] (Table 1). In ovarian cancer, patients who were prior recipients of solid organ transplants or who were receiving immunosuppressive therapies were not excluded from randomized bevacizumab trials, but those with pre-existing uncontrolled hypertension or renal dysfunction based on serum creatinine ≥ 1.6 mg/dL or proteinuria > 1 g per 24 h were excluded [19,20,21]. Similarly, in other large randomized angiogenesis inhibitor studies across other tumor sites, prior solid organ transplant or use of immunosuppressants is not an exclusion criterion, aside from studies involving immune checkpoint inhibitors [22]. Identification of patients enrolled in large angiogenesis inhibitor trials who had received prior transplants could potentially make for an interesting post-hoc pooled analysis.

Similarly, reports on angiogenesis inhibition in solid organ transplant patients remain scant in the literature, as highlighted by a review on bevacizumab toxicity by Fenoglio et al [9]. Musri et al. reported a case of colorectal cancer post-renal transplantation with baseline proteinuria, which significantly worsened on administration of intravenous 5-fluorouracil, irinotecan, oxaliplatin, and bevacizumab [23]. Cheungpasitporn et al. described two cases with renal allograft dysfunction following administration of intravitreal bevacizumab, aflibercept, or ranibizumab [24]. Doses of anti-angiogenics were lower but not specified within this report. Although neither case proved causality with anti-VEGF therapy, one was diagnosed with phospholipase A2 receptor-negative membranous nephropathy, and the second revealed acute and chronic antibody-mediated rejection with glomerular thrombi and transplant glomerulopathy. Jonkers and Buren reported a case of worsening IgA nephropathy presenting with nephrotic-range proteinuria post-renal transplantation on sorafenib [25]. These reports highlight the potential severe nephrotoxicity known to be associated with angiogenesis inhibitors; however, there remain few documented positive experiences with angiogenesis inhibitor use in the post-transplant setting. Given the prevalence of nephrotoxicity with these agents, these considerations are particularly prudent for renal transplant recipients, but reports in other organ transplants remain similarly scarce.

### 3.2. Medication Interactions

Solid organ transplant recipients frequently take maintenance immunosuppressive agents, including but not limited to corticosteroids, calcineurin inhibitors, anti-proliferative agents, and mTOR inhibitors, which are associated with various complications and drug interactions (Table 2).

In the case presented, tacrolimus levels were measured every few months to be within the therapeutic range; this is significant as potential drug interactions between transplant medications and angiogenesis inhibitors involve pharmacokinetic and pharmacodynamic interactions. Pharmacokinetic interactions typically occur due to cytochrome P450 enzyme (CYP) and P-glycoprotein (P-gp) drug transport systems [26,27,28], and risk of competitive metabolism as substrates for the same enzyme or transporter may increase serum levels. Axitinib and sorafenib are CYP3A4 and P-gp substrates, and cabozantinib and pazopanib are substrates and inhibitors of both enzyme systems [29,30,31,32]. Drug interactions are well-described for CYP3A4 substrates cyclosporine, tacrolimus, and sirolimus, with metabolic inhibition leading to increased immunosuppressant concentrations (~20%) [28]. Pharmacodynamic interactions primarily concern cumulative toxicities between these two classes (Table 2) [26,27,33,34]. Interestingly, Onodera et al. reported upon a case of metastatic colorectal cancer post-renal transplant where a patient was administered five cycles of 5-fluorouracil, oxaliplatin, and bevacizumab where although severe proteinuria occurred, serum tacrolimus levels were not affected throughout the course of treatment [35]. This report remains one of the only cases in the literature that demonstrates stability of immunosuppression whilst on bevacizumab post-transplant.

### 3.3. Long-Term Adverse Events

One of the leading causes of morbidity and mortality in solid organ transplant recipients is malignancy, most commonly non-melanomatous skin cancers [36]. Other malignancies such as colorectal, kidney, and cervical cancers are also prevalent in the post-transplant context, and angiogenesis inhibitors such as bevacizumab are commonly used in metastatic disease [18] (Table 1). Surveillance recommendations within transplant recipients are variable across the globe due to a paucity of robust screening trials [37]. Other long-term complications associated with organ transplantation and prolonged immunosuppressant use include cardiovascular disease, diabetes mellitus, hypertension, and infection associated with cytopenia. In the patient presented, adverse events have not outweighed benefits of ongoing treatment, but this will need close monitoring given the risk of overlapping toxicities as long-term adverse event data remain limited [3,38].

## 4. Conclusions

Whilst there are minimal data justifying that bevacizumab or other angiogenesis inhibitors are unsafe in the post-transplant setting, there is similarly scarce literature demonstrating safe administration, as in the patient presented. As life expectancy continues to improve with increasing indications for transplantation, long-term risks for malignancy with prolonged immunosuppression are increasingly relevant as a cause of mortality in solid organ transplant recipients. In patients who have undergone renal transplantation, careful consideration of treatment options with risk of nephrotoxicity and close monitoring remains paramount.

Although treating oncologists should remain vigilant about potential drug interactions and overlapping toxicities, these are not necessarily contraindications for agents such as bevacizumab. Treatment decisions should consider the best available evidence, and collating information about toxicity and tolerance from randomized trials and post-approval Phase IV studies would provide detailed information from at-risk subgroups. This calls for a stratified, inclusive approach to allow enrolment of those with chronic diseases and comorbidities in prospective trials, allowing objective assessment of the risk–benefit ratio.

## Figures and Tables

**Table 1:** Food and Drug Administration approved indications for bevacizumab [18].

Cancer	Stage	Usage
Colorectal	Metastatic, first-line	5 mg/kg every two weeks with bolus IFL 10 mg/kg every two weeks with FOLFOX4
	Metastatic, recurrent after first-line bevacizumab-containing regimen	5 mg/kg every two weeks, or 7.5 mg/kg every three weeks with fluoropyrimidine–irinotecan, or fluoropyrimidine–oxaliplatin-based chemotherapy
Non-squamous, non-small-cell lung	Unresectable, locally advanced, recurrent, or metastatic	15 mg/kg every three weeks with carboplatin and paclitaxel
Glioblastoma	Recurrent	10 mg/kg every two weeks
Renal cell	Metastatic	10 mg/kg every two weeks with interferon-alfa
Cervical	Persistent, recurrent, or metastatic	15 mg/kg every three weeks with paclitaxel and cisplatin, or paclitaxel and topotecan
Epithelial ovarian, fallopian tube, or primary peritoneal	III or IV, following surgical resection	15 mg/kg every three weeks with carboplatin and paclitaxel for up to six cycles, followed by 15 mg/kg every three weeks as a single agent for up to 22 cycles
	Recurrent, platinum-sensitive	15 mg/kg every three weeks with carboplatin and either paclitaxel (6–8 cycles) or gemcitabine (6–10 cycles) followed by 15 mg/kg every 3 weeks as a single agent
	Recurrent, platinum-resistant	10 mg/kg every two weeks with paclitaxel, pegylated liposomal doxorubicin, or topotecan given weekly 15 mg/kg every three weeks with topotecan every three weeks
Hepatocellular	Unresectable or metastatic, first-line	15 mg/kg with atezolizumab every three weeks

Abbreviations: mg/kg = milligrams per kilogram; IFL = infusional fluoropyrimidine; FOLFOX4 = 5-fluorouracil, folic acid, and oxaliplatin.

**Table 2:** Drug–drug interactions between post-transplant immunosuppressive medications and angiogenesis inhibitors.

Transplant Medication	Potential Interactions with Anti-Angiogenesis Agents [26,27,28,29,30,31,32,33,34]
**Cyclosporine**	Increased cyclosporine levels and subsequent toxicity due to CYP3A4 and P-gp-mediated drug interactions (e.g., cabozatinib, axitinib, pazopanib, sorafenib, sunitinib)
**Tacrolimus**	Increased tacrolimus levels and subsequent toxicity due to inhibition of or competition with CYP3A4 metabolism and P-gp-mediated transport (e.g., cabozatinib, axitinib, pazopanib, sorafenib, sunitinib) Additive impairment of the renal function (e.g., cediranib, axitinib, pazopanib) Increased risk of QT prolongation with other agents that prolong the QT interval (e.g., cabozantinib, pazopanib, sorafenib, sunitinib)
**Mycophenolate mofetil**	Exaggerated leukopenia (e.g., ramucirumab, bevacizumab, sunitinib)
**Azathioprine**	Exaggerated leukopenia (e.g., ramucirumab, bevacizumab, sunitinib)
**Sirolimus**	Increased tacrolimus levels and subsequent toxicity due to inhibition of or competition with CYP3A4 metabolism and P-gp-mediated transport (e.g., cabozatinib, axitinib, pazopanib, sorafenib, sunitinib) Additive impairment of the renal function (e.g., cediranib, axitinib, pazopanib) Additive impairment of wound healing
**Everolimus**	Increased everolimus levels and subsequent toxicity due to inhibition of CYP3A4 metabolism and P-gp-mediated transport (e.g., cabozantinib, pazopanib) Additive impairment of the renal function (e.g., cediranib, axitinib, pazopanib) Additive impairment of wound healing
**Corticosteroids**	Competitive CYP3A4 metabolism (e.g., prednisone) with other CYP3A4 substrates (e.g., cabozantinib, axitinib, pazopanib, sorafenib, sunitinib)

Abbreviations: CYP3A4 = cytochrome P450 3A4; P-gp = P-glycoprotein.

## Data Availability

Not applicable.

## Abbreviations

VEGFR	vascular endothelial growth factor receptor
EGFR	epidermal growth factor receptor
FGFR	fibroblast growth factor receptor
PDGFR	platelet-derived growth factor receptor
SCF	stem cell factor
PI3K	phosphoinositide 3-kinase
AKT	protein kinase B
mTOR	mammalian target of rapamycin
RET	RET proto-oncogene
AXL	AXL receptor tyrosine kinase
TAH–BSO	total abdominal hysterectomy and bilateral salpingo-oophorectomy
Ca125	cancer antigen 125
U/ml	units per milliliter
AKI	acute kidney injury
CYP	cytochrome P450
HGSOC	high-grade serous ovarian carcinoma
FOLFOX4	5-fluorouracil, leucovorin, and oxaliplatin
IFL	irinotecan, leucovorin, and 5-fluorouracil
P-gp	P-glycoprotein
